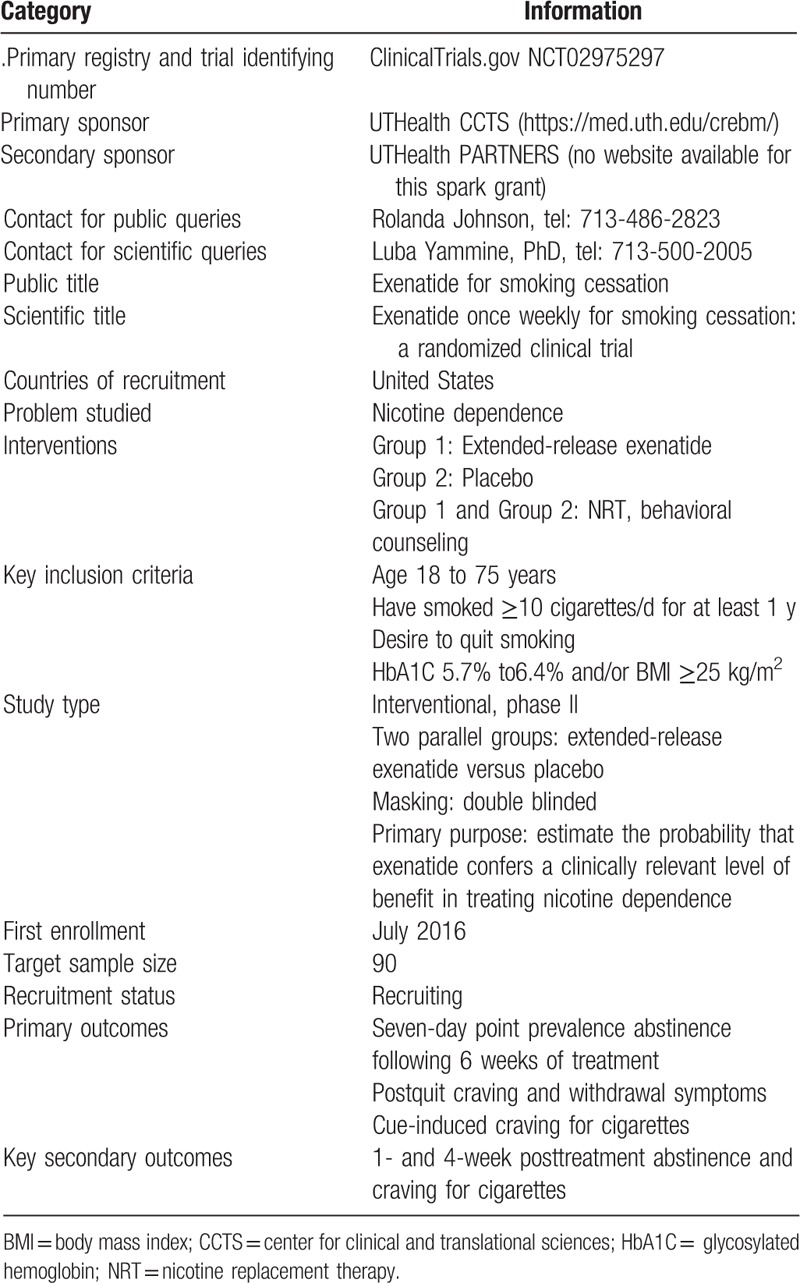# Exenatide once weekly for smoking cessation: study protocol for a randomized clinical trial

**DOI:** 10.1097/MD.0000000000009567

**Published:** 2018-01-12

**Authors:** Luba Yammine, Thomas R. Kosten, Paul M. Cinciripini, Charles E. Green, Janet C. Meininger, Jennifer A. Minnix, Thomas F. Newton

**Affiliations:** a University of Texas Health Science Center at Houston; b Baylor College of Medicine; c University of Texas MD Anderson Cancer Center, Houston, Texas; d Mind Springs Health, Grand Junction, Colorado.

**Keywords:** craving, glucagon-like peptide-1, smoking, smoking abstinence, study protocol, withdrawal

## Abstract

**Background:**

Cigarette smoking is the greatest preventable cause of morbidity and premature mortality in the United States. Approved pharmacological treatments for smoking cessation are marginally effective, underscoring the need for improved pharmacotherapies. A novel approach might use glucagon-like peptide-1 (GLP-1) agonists, which reduce alcohol and drug use in preclinical studies. GLP-1 is produced in the intestinal L-cells and in the hindbrain. The peptide maintains glucose homeostasis and reduces food intake. Several GLP-1 agonists are used clinically to treat type 2 diabetes and obesity, but none have been tested in humans to reduce smoking.

**Aims:**

We will examine whether extended-release exenatide reduces smoking, craving, and withdrawal symptoms, as well as cue-induced craving for cigarettes.

**Methods:**

We will enroll prediabetic and/or overweight treatment seeking smokers (n = 90) into a double-blind, placebo-controlled, randomized clinical trial. Participants will be randomized in a 1:1 ratio to receive exenatide or placebo. All participants will receive transdermal nicotine replacement therapy (NRT) and behavioral counseling. Abstinence from smoking (verified via expired CO level of ≤5 ppm), craving (Questionnaire of Smoking Urges score), and withdrawal symptoms (Wisconsin Scale of Withdrawal Symptoms score) will be assessed weekly during 6 weeks of treatment and at 1 and 4 weeks posttreatment. Cue-induced craving for cigarettes will be assessed at baseline and at 3 weeks of treatment following virtual reality exposure.

**Expected outcomes:**

We hypothesize that exenatide will increase the number of participants able to achieve complete smoking abstinence above that achieved via standard NRT and that exenatide will reduce craving and withdrawal symptoms, as well as cue-induced craving for cigarettes.

## 1 INTRODUCTION

Cigarette smoking is the leading cause of preventable mortality in the United States, producing over 400,000 deaths annually.^[1]^ Moreover, an additional 50,000 deaths occur each year among nonsmokers who are exposed to secondhand smoke. Over 16 million Americans suffer from chronic diseases caused by smoking, and these diseases cost the US $170 billion in direct medical care and over $156 billion in lost productivity annually.^[1]^

The first-line pharmacological therapies for smoking cessation include nicotine replacement therapy (NRT), bupropion hydrochloride, and varenicline tartrate. Nearly 50 million Americans smoke cigarettes, and although many report wanting to quit, the 12-month abstinence rates with the use of these therapies range from 22% to 33%.^[1]^ The relatively modest long-term improvements in abstinence rates underscore the importance of developing improved therapeutic approaches to assist smoking cessation.^[2,3]^ One innovative approach is based on animal studies showing that glucagon-like peptide-1 (GLP-1) receptor agonists attenuate rewards induced by alcohol,^[4,5]^ cocaine,^[6,7]^ amphetamine,^[6,8]^ and most relevant here, nicotine.^[9]^ In mice, treatment with a GLP-1 agonist at a dose with no other behavioral effects attenuated nicotine-induced locomotor stimulation, nucleus accumben's dopamine release, and reduced the expression of nicotine conditioned place preference.^[9]^

GLP-1 is produced endogenously in the intestinal L-cells and in the hindbrain nucleus tractus solitarius^[10–13]^ in response to nutrient ingestion. Acting primarily via GLP-1 receptors in hypothalamus and brain stem, GLP-1 regulates glucose homeostasis via increasing the secretion of insulin^[14]^ and suppressing the secretion of glucagon.^[15,16]^ GLP-1 also reduces food intake^[17,18]^ in part by reducing appetite.^[19–21]^ A GLP-1 agonist exenatide is food and drug administration (FDA) approved for the treatment of type 2 diabetes mellitus (DM), and a related GLP-1 agonist liraglutide is FDA approved for the treatment of obesity in patients with or without DM. GLP-1 receptors are expressed in many brain regions. In addition to the hypothalamus and brain stem, GLP-1 receptors are expressed throughout the mesolimbic dopamine system,^[22]^ and GLP-1 containing neurons extend directly into the ventral tegmental area and nucleus accumbens^[23]^ – areas that are intimately associated with the regulation of reward. These data suggest that the role of GLP-1 extends beyond its glucose regulatory effects to reward regulation and that the GLP-1 system may represent a target for pharmacological treatment of addictive behaviors.^[17,18]^

Our objectives are to evaluate, for the first time, the impact of treatment with extended-release injectable exenatide on smoking abstinence and cue-induced craving for cigarettes among prediabetic and/or overweight adults. In addition, to obtain preliminary evidence for a larger follow-up study that will evaluate the mediating effects of craving and withdrawal symptoms on abstinence in smokers treated with exenatide, we will assess the impact of exenatide on postquit craving and withdrawal symptoms – proximal factors that can covary with and predict future abstinence.^[24–27]^

## 2 METHODS

### 2.1 Study design and rationale

#### 2.1.1 Study design

This double-blind, placebo-controlled, randomized clinical trial will be conducted in a research clinic associated with the academic center. The participants will be 90 adult smokers who have prediabetes (Type 2, glycosylated hemoglobin [HbA1C] = 5.7–6.4%) and/or are overweight (Body mass index≥25 kg/m^2^).^[28–31]^ The participants will be randomized in a 1:1 ratio to receive exenatide or placebo. All participants, including those in the active medication group and those in the placebo group, will receive transdermal NRT (nicotine patches) and individual behavioral smoking cessation counseling. Quit date will be set following 2 weeks of exenatide/placebo treatment because we anticipate that it will take 2 weeks for the effects of exenatide to emerge.^[32,33]^ Smoking abstinence will be evaluated via self-report and confirmed during weekly clinic visits via breath carbon monoxide (CO) measurements.

Rationale: Administration of extended-release exenatide (2 mg) results in therapeutic concentrations of >50 pg/mL in approximately 2 weeks, and the steady-state concentrations of approximately 300 pg/mL are achieved within approximately 4 to 6 weeks.^[32,33]^ Based on these pharmacokinetics, we selected to set the quit date following 2 weeks of exenatide treatment and to evaluate the primary outcomes following a total of 6 weeks of treatment. This is the first study to evaluate the effect of exenatide treatment on smoking outcomes; therefore, the optimal time points for quit date and assessment of the primary outcomes cannot be predicted with certainty. To mitigate this concern, we will evaluate the trajectory of quit attempts and abstinence throughout the duration of the study.

### 2.2 Study aims

#### 2.2.1 Primary aims

Aim 1: To examine the effect of extended-release exenatide treatment on CO confirmed self-reported smoking abstinence in the study participants following a quit attempt.

Hypothesis 1.a.: Exenatide will increase the rate of complete smoking abstinence measured by 7-day point prevalence abstinence (self-reported and biochemically verified via expired CO levels) following 6 weeks of treatment.

Hypothesis 1.b.: Exenatide-treated participants will show differential change over time in the probability of abstinence as measured by 7-day point prevalence during the 6-week treatment.

Aim 2: To examine the effect of extended-release exenatide treatment on postquit craving and withdrawal symptoms in the study sample.

Hypothesis 2.a.: Exenatide will reduce postquit craving (as measured by the Questionnaire of Smoking Urges [QSU] score) and withdrawal symptoms (as measured by the Wisconsin Smoking Withdrawal Scale score) following 6 weeks of treatment.

Hypothesis 2.b.: Exenatide-treated participants will show differential reduction in postquit craving and withdrawal during the 6-week treatment.

Aim 3: To examine the effect of extended-release exenatide on cue-induced craving for cigarettes.

Hypothesis: Exenatide-treated participants will display lower craving for cigarettes (as measured by the QSU score) following virtual reality (VR) exposure.

#### 2.2.2 Secondary aim

Aim 1: To examine the effect of the 6-week extended-release exenatide treatment on 1- and 4- week posttreatment abstinence from smoking and craving for cigarettes in the study participants.

Hypothesis: Exenatide will increase the rate of complete smoking abstinence measured by 7-day point prevalence abstinence (self-reported and biochemically verified via expired CO levels) and reduce postquit craving (as measured by the QSU score) at 1- and 4-weeks posttreatment.

### 2.3 Participants

Participants will be 90 men and women between ages 18 and 75 years who have been daily smokers for at least 1 year, are currently smoking ≥10 cigarettes a day and desire to quit smoking, and are not currently using any pharmacotherapy for smoking cessation. Participants must have HbA1C levels between 5.7% and 6.4% and/or a body mass index of ≥25 kg/m^2^.

To preserve high internal validity and minimize the risk of adverse events, the following exclusion criteria will be applied: psychotic or bipolar disorder, or mood disorder with psychotic features (existing diagnosis or as determined by the structured interview); moderate to high risk of suicidality; psychoactive substance abuse or dependence (excluding nicotine dependence) within the past 3 months; personal or family history of medullary thyroid carcinoma or multiple endocrine neoplasia syndrome type 2; type 1 diabetes mellitus; current use of oral or injectable glucose lowering medications; severe cardiovascular disease (history of myocardial infarction, life-threatening arrhythmia, or worsening angina pectoris); active temporomandibular joint disease; severe gastrointestinal disease (i.e., severe gastroparesis); previous history of pancreatitis or risk of pancreatitis; creatinine clearance <30; previous medically adverse reaction to study the medication, nicotine, or menthol; women who are currently pregnant or lactating, or of childbearing potential and are not using medically accepted forms of contraception.

### 2.4 Procedures

This study is funded by the University of Texas Health Science Center at Houston (UTHealth) Center for clinical and translational sciences Scholar Award and by the UTHealth PARTNERS Research Award. The institutional review board (IRB) has approved the study. The study is registered on clinicaltrials.gov (trial identifier N02975297).

#### 2.4.1 Screening

Participants will be recruited using various strategies, including flyers, posters, and newspaper ads. When the potential participants call the provided telephone number, the requirements and the procedures of the study will be explained, and a brief prescreen to determine initial eligibility will be administered. The prescreen interview includes questions concerning medical and psychiatric history, psychoactive substance use, and smoking history. Individuals who appear eligible based on the initial prescreen will be invited for a face-to-face interview (screening visit).

The screening visit will begin with the completion of the informed consent which will be obtained by the study principal investigator (PI). Informed consent procedures will include details of the study, potential risks, study timeline, and voluntariness for participation and dropping out. Following completion of the informed consent process, each participant will provide his or her demographic and detailed medical history, followed by validation of the smoking history, current smoking (assessed by a CO monitor, Micro^+^ Smokerlyzer, Williamsburg, Virginia), and assessment of nicotine dependence. Subsequently, each participant will undergo a mini international neuropsychiatric interview (MINI) neuropsychiatric interview assessment and a brief physical exam, including assessment of vital signs and collection of blood samples for evaluation of HbA1C, complete blood count, chemistry, liver function, renal function, and amylase and lipase levels. A urine-based pregnancy test for women of childbearing potential will be also completed.

#### 2.4.2 Randomization

The research coordinator will randomize participants in a 1:1 ratio into exenatide and placebo groups via a computer-generated random number sequence, using blocks of 4. Randomization will be performed on the day of a participant's first study visit.

#### 2.4.3 Study medication/placebo

##### 2.4.3.1 Exenatide

Exenatide will be purchased commercially as Bydureon for subcutaneous injection. The incidence of hypoglycemia with the use exenatide is low because the release of insulin following administration of exenatide is glucose mediated. Although there have been questions about the safety of incretins such as exenatide, as treatment may be associated with increased rates of pancreatitis, a meta-analysis of 60 studies (n = 353,639), consisting of 55 randomized controlled trials (n = 33,350) and 5 observational studies (3 retrospective cohort studies, and 2 case–control studies; n = 320,289) has shown that incretin treatment was not associated with increased rates of pancreatitis.^[34]^ Ongoing postmarketing monitoring is also underway. In addition, data from rodent studies suggest that GLP-1 agonists may be associated with an increased risk of thyroid C-cell hyperplasia and C-cell tumors; however, experiments with monkeys did not show proliferation of C-cells in thyroid gland after chronic administration of GLP-1 agonists. Longitudinal data from clinical trials have not demonstrated a causal association between GLP-1 agonists and thyroid C-cell pathology over a 2-year period.^[35]^ To address the concerns regarding the risk of pancreatitis and/or thyroid cancer associated with GLP-1 agonists, potential participants with abnormal levels of pancreatic enzymes, history of pancreatitis, or at risk for pancreatitis, as well as those with personal and/or family history of thyroid C-cell tumor or multiple endocrine neoplasia syndrome type 2 will be excluded from participation in this study.

##### 2.4.3.2 Placebo

Sterile saline will serve as the placebo for exenatide.

##### 2.4.3.3 Criteria for withholding study treatment

Subsequent doses of exenatide will not be administered if any of the following occur: hypoglycemia, hypersensitivity reaction, anaphylaxis, nephrotoxicity, pancreatitis, or severe injection site reaction or the study PI believes that there may be any reason to withhold exenatide.

##### 2.4.3.4 Criteria for participant discontinuation following study initiation

Participants will be discontinued from the study participation if they are unable to comply with the study procedures or if they meet discontinuation criteria because of side-effects/adverse events associated with the study treatment.

#### 2.4.4 Stopping criteria

The frequency of the treatment-related adverse events will be monitored. If more than 10% of participants entering the study experience treatment-related adverse event, the study will be halted if indicated by the pattern and severity of adverse events.

#### 2.4.5 NRT (nicotine patches)

NRT patches, 21 mg (generic) will be purchased commercially. The patches will be dispensed during clinic visits in the amount sufficient for 1 week of use.^[36]^ NRT patches are an over-the-count product with a proven safety record. The patches are contraindicated in persons with hypersensitivity to nicotine or menthol, those with severe cardiovascular disease (i.e., history of myocardial infarction, life-threatening arrhythmia, or worsening angina pectoris), and those with temporomandibular joint disease. To address these concerns, individuals with these conditions will be excluded from participation in the study.

#### 2.4.6 Smoking cessation counseling

All participants will receive brief individual behavioral smoking cessation counseling as is the recommended standard for use with pharmacotherapy.^[37]^The counseling protocol is manual driven, has been used in several previous studies,^[38–40]^ and consists of 6 in-person sessions (on the days when the participants come to clinic to receive exenatide/placebo treatment) and 2 brief supportive phone calls (1-day prequit and 3 days postquit), lasting 10 to 15 minutes each, spanning the 6 week active treatment phase. Counseling content follows the previous study^[40]^ and briefly involves preparation for quitting, identification of high-risk situations for smoking, development of coping skills and direct support before and after the quit date, motivational intervention for keeping or resetting a quit date, management of withdrawal symptoms, and medication compliance. Counseling will be provided by a family nurse practitioner with a PhD degree, who is a certified tobacco treatment specialist and who has been trained by smoking cessation clinicians who have extensive experience from the previous trials in training and monitoring the integrity of the counseling protocol, including recording time and content of each session^[29–31]^ At the conclusion of active treatment, participants will be offered referral for ongoing cessation counseling (quitline).

#### 2.4.7 Virtual reality (cue-induced craving for cigarettes)

To assess baseline withdrawal symptoms and craving for cigarettes participants will complete the Wisconsin Scale of Withdrawal Symptoms (WSWS, described in 2.4.7.) and the QSU (described in 2.4.7). Subsequently, participants will take part in a VR session that is viewed using a head-mounted device (NIVS SX-60, NIVS, Reston, Virginia) with a head tracker (Ic3 inertia cube, Intersense) connected to a desktop computer. Participants are exposed to VR neutral cues (nature scenes), followed by series of VR smoking-related cues. VR smoking cues are personalized to match each participant's preferred brand of cigarettes. Similarly, the style of music played in the VR party contexts is also adjusted based on participant preference. Cue reactivity will be assessed within the VR environment immediately after each VR scenario with the aid of a gamepad to answer craving-related questions (using a visual analog scale of 0–100). VR neutral context consists of a nature video; VR active paraphernalia context consists of proximal smoking-related objects (packs of cigarettes, ashtrays, bottles of alcohol) placed on tables around the room; and VR party context consists of similar smoking cues and includes interactions with avatars at the party where cigarettes are offered, drinks are poured, and conversations are held while people light up cigarettes. Exposure to each VR environment (neutral, paraphernalia, party) lasts approximately 3 minutes and follows a timed path where at certain points participants are visually directed for a few seconds to pay attention to nonsmoking or active cues to standardize some of the VR exposure within the environment. This VR paradigm was used in a previous study that showed that self-reported levels of ‘craving’ (*P* < .01) and ‘thinking about cigarettes’ (*P* < .0001) were significantly greater after exposure to the active cues versus nonsmoking cues and that there were significant positive correlations between self-reported craving prior to the VR session and craving induced by active VR cues (*P* < .01).^[41]^

#### 2.4.8 Data collection and measures

The MINI Neuropsychiatric Interview will be administered during screening to determine whether study participants meet the diagnostic and statistical manual-5 criteria for nicotine-use disorder and to rule out presence of exclusionary conditions noted in 2.3.^[42]^

Nicotine dependence will be assessed during screening using the Fagerstrom test for nicotine dependence (FTND).^[43]^ The FTND is a valid and reliable 6-item questionnaire that evaluates the quantity of cigarette consumption, the compulsion to use, and dependence.

Baseline smoking status will be assessed during screening via self-reported smoking of ≥10 cigarettes/day for >1 year and with recent smoking confirmed by an expired air CO level of ≥10 ppm.

Self-reported abstinence from smoking will be confirmed via time-line follow back procedures.^[44]^ Biochemical verification of abstinence (expired CO level of ≤5 ppm) will be performed during clinic visits.

Withdrawal symptoms will be assessed using WSWS.^[45]^ WSWS is a valid and reliable questionnaire which includes subscales of anger, anxiety, concentration, craving, hunger, sadness, and sleep.

Craving for smoking will be evaluated using the QSU,^[46]^ a 32-item scale that assesses the intention and desire to smoke and an anticipation of relief from the withdrawal-associated negative affect.

Positive and negative affect will be assessed using the positive and negative affect schedule,^[47]^ a 20-item questionnaire that asks participants to rate the extent to which they experience positive (i.e., excited, inspired, proud) or negative (i.e., irritable, distressed, upset) emotions.

#### 2.4.9 Study visits

Following determination of the study eligibility, qualified participants will return to clinic for 6 weekly visits. At each visit smoking during the previous week will be ascertained via self-report and recent smoking will be verified using the CO monitor; vital signs and finger-stick blood glucose will be measured; and the participants’ well-being, including any side-effects/adverse events will be assessed. The participants will also complete a number of assessments (described in detail in 2.4.7.) and receive smoking cessation counseling (described in detail in 2.4.5.) and NRT for the upcoming week. To facilitate adherence to the study medication, participants will be administered the study medication/placebo (described in detail in 2.4.3.) during each study visit. Cue-induced craving for cigarettes will be assessed following VR exposure during visit 1 (baseline) and visit 3 (described in detail in 2.4.6.). Upon the completion of the treatment phase of the study, the participants who were abstinent following 6 weeks of treatment will be contacted via telephone at 1- and 4-weeks posttreatment to ascertain continued abstinence from smoking. Those who report being abstinent, will be invited for in-person visit for the biochemical verification of abstinence. Please refer to Table 1 for the study timeline.

**Table 1:**
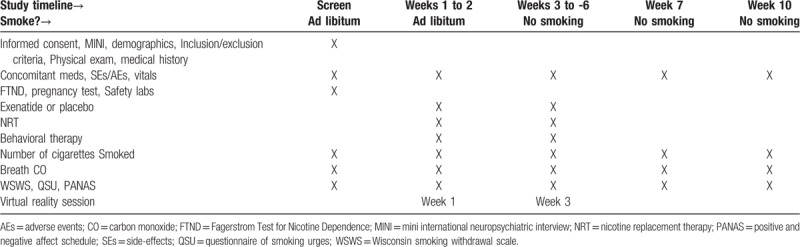
Study procedures.

#### 2.4.10 Outcome measures

The primary outcomes for this study are 7-day point prevalence abstinence (self-reported and biochemically verified via expired CO level of ≤5 ppm) following 6 weeks of treatment, postquit craving (as measured by the QSU score), and withdrawal symptoms (as measured by the WSWS score) following 6 weeks of treatment, and cue-induced craving for cigarettes (as measured by QSU score) following VR exposure. The key secondary outcomes are 7-day point prevalence abstinence (self-reported and biochemically verified via expired CO level of ≤5 ppm) and postquit craving (as measured by the QSU score) at 1- and 4-weeks posttreatment.

#### 2.4.11 Safety assurances

##### 2.4.11.1 Medical monitoring

Vital signs will be assessed at each visit. Blood glucose levels will be assessed prior to each dose of exenatide. The PI will halt exenatide administration if stopping criteria are met. The dose of exenatide that we will use is the standard dose that is currently used clinically; therefore, we expect no medical issues to arise. Should a participant become unable to cooperate with study procedures, he or she will be replaced.

##### 2.4.11.2 Adverse event assessment and management

Participants’ well-being will be assessed at each visit. Participants will be asked if they are experiencing any discomfort indicating potential side-effects. Spontaneously reported symptoms or complaints will be recorded and reported as required if events are classified as serious.

#### 2.4.12 Blinding

The care providers, outcome assessors, and the statistician performing the analysis will remain blinded to the participants’ assigned conditions (exenatide versus placebo) throughout the study. Participants will not be aware as to whether they were assigned to the active or control condition.

#### 2.4.13 Estimated timeline of the study

Recruitment for this study began in July 2016 and is anticipated to continue through June 2019. Anticipated recruitment is estimated to be approximately 4 new participants per month.

#### 2.4.14 Participant retention and remuneration

To facilitate retention, participants will be remunerated according to the following increasing payment schedule: Screening visit – $10; study visit 1 – $10; study visit 2 – $15; study visit 3 – $20; study visit 4 – $25; study visit 5 – $30, study visit 6 – $50; posttreatment visit 1 – $10; posttreatment visit 2 – $10. Participants will receive compensation at the end of each visit. For participants who decide to withdraw from the study, we will inquire about the reasoning for their decision and will report this qualitatively in the manuscript.

#### 2.4.15 Data entry and storage

Data will be entered into password-protected excel files by the study staff. The study staff received extensive training in good clinical practices to ensure that all data collected accurately reflect source documents. The study staff will thoroughly check data from clinical charts against source documents. Twenty percent of the outcome data will be double entered. In the event of major discrepancies, outcome data will receive 100% source document verification. Only investigators will have access to the data. All data generated or analyzed during this study will be available from the corresponding author on request.

#### 2.4.16 Privacy and confidentiality

All precautions will be taken to safeguard participants’ privacy and prevent breach in confidentiality. The preintake screening visit will be administered in private with the utmost sensitivity to the participant. Personal questions about medical history, lifestyle, and drug use will be asked in a private room such that no one apart from the study staff will know the answers. Participant information that is entered into the database will be assigned a unique code to help prevent a breach of confidentiality. All information, which includes the preintake screening, clinical data, and questionnaires, will be kept confidential and stored in a highly secure database system. No personal identifying information will be stored in this system. Access to this database is limited to authorized investigators.

#### 2.4.17 Data monitoring

The study PI and research team will monitor data on an ongoing basis. In addition, a data and safety monitoring board (DSMB) will oversee the ongoing progress of the study. The board consists of 2 physicians, a psychologist, and a biostatistician who are not affiliated with the study or the sponsor. DSMB evaluations will be conducted on the annual basis. In addition, the DSMB will perform an end-of-study evaluation.

#### 2.4.18 Protocol modifications

The PI will receive IRB approval before initiating any changes, including those required by the sponsor, which would affect study participants, such as changes in methods or procedures, numbers or kinds of the study participants, or revisions to the informed consent document or procedures. All protocol revisions will be submitted to the primary sponsor of the research.

## 3 DATA ANALYSIS

### 3.1 Statistical methods

Whether GLP-1 agonists confer benefit in treating nicotine dependence has not been tested in clinical trials. Frequent statistics that are based on dichotomous null hypothesis testing (i.e., estimating the probability of observing the data, or data more extreme, given that the null hypothesis is true) would be less informative in the context of this early-phase project. We, therefore, selected a Bayesian approach to data analysis. Bayesian probability estimates incorporate prior information about plausible parameter values with the observed data, forming the posterior distribution and thereby, allow estimates of the probability that the true value of the parameter falls in some range. The Bayesian approach will thus allow us to bet on the alternative hypothesis and ascertain the probability that the treatment confers some level of benefit, given the observed data.^[48]^ According to the FDA, Bayesian statistics offer improved methodological efficiency for early-phase projects aimed to ‘repurpose’ existing therapies for new indications.^[49–51]^ Decision-making based on an initial trial of a compound for a new indication is assisted by estimates of the probability of an effect of some specified magnitude, even with small sample sizes.^[52,53]^

### 3.2 Statistical analysis

An intent-to-treat approach will impute those who drop out of the study being counted as continuing to smoke. Demographic and baseline characteristics of the study participants by group that show correlations with both treatment group and outcomes may be potential confounders and result in 2 sets of analyses, those including and those excluding the salient demographic or baseline characteristic to ascertain the degree to which this is the case. If these analyses suggest evidence of confounding, we will report both unadjusted estimates of treatment effect and estimates statistically adjusted using covariance analyses for the potentially confounding factors. For retention, group differences in the time to dropout over the 6-week study period as a function of treatment will use Cox proportional hazards survival analysis. Data analyses will use generalized linear and multilevel models. Binomial regression models (with a log link to permit estimates of relative risks) will be used to assess the effect of treatment on the 7-day point prevalence abstinence following 6 weeks of treatment and 1- and 4-weeks posttreatment. Multilevel models will be used to assess the effect of treatment on craving and cue-induced craving for cigarettes (QSU score), and withdrawal symptoms (WSWS score). Lastly, multilevel models will be used to examine the trajectory of abstinence throughout the course of the study. Vague, neutral priors provide a basis for initially evaluating the trial results: for linear Cox proportional hazards and logistic regression, priors for coefficients will take the form ∼normal (μ = 0, σ^2^ = 1 × 10^6^) in the linear, log-hazard, and log-relative risk scales. Priors for error or dispersion terms will use ∼gamma (a = 0.001, b = 0.001) and ∼uniform (11,000) for level 1 and 2 effects, respectively. We will investigate the robustness of the resulting posterior distributions using a variety of neutral and pessimistic priors representing vague, weakly informative, and skeptical informative perspectives. Informative priors will use extant information (e.g., meta-analytic findings^[54]^ for smoking outcomes). Evaluation of posterior distributions will permit statements regarding the probability that effects of varying magnitudes exist, given the data.

### 3.3 Dissemination of findings

After the study is completed, the results of the analyses will be published. No personal information will be included in the findings.

## 4 DISCUSSION

The objective of this early-phase project is to estimate the probability that exenatide confers a clinically relevant level of benefit in treating nicotine dependence. If our hypotheses are confirmed, the results of this project would provide strong preliminary data in support of a definitive follow-up study. In a follow-up study we would assign study participants to placebo and exenatide with quit dates at 2 and 6 weeks of treatment to determine the onset of clinical effects. We would then increase the study duration to at least 12 weeks. This would enable us to clarify the length of time needed to achieve the therapeutic levels and would provide a clearer test of efficacy. We will also examine the mediating effects of craving, withdrawal symptoms, and gustatory sensation on abstinence, which would elucidate the mechanisms by which GLP-1 agonists enhance smoking abstinence. Last, we will examine other important abstinence-related outcomes, such as weight and glycemia, among others.

Overall, there are at least 2 aspects of this study that are highly important:

1) This is the first clinical study to evaluate exenatide as potential treatment for nicotine dependence. If this treatment is effective, it could represent a new approach to assist smoking cessation. In addition, because exenatide is unrelated to other smoking cessation therapies, it could be combined with other treatments, such as NRT or varenicline, to create an effective ‘quit package.’
2) If exenatide is effective as treatment for nicotine dependence, smokers with DM would require only 1 treatment modality for glucose control and smoking cessation.

Trial registration data.

**Figure:**